# Transposable elements and xenobiotic resistance

**DOI:** 10.3389/finsc.2023.1178212

**Published:** 2023-06-12

**Authors:** Richard H. ffrench-Constant

**Affiliations:** Center for Ecology and Conservation, University of Exeter in Cornwall, Penryn, United Kingdom

**Keywords:** insecticide, resistance, transposable element, transposon, insect

## Abstract

Transposable elements or TEs are well known drivers of adaptive change in plants and animals but their role in insecticide resistance remains poorly documented. This review examines the potential role of transposons in resistance and identifies key areas where our understanding remains unclear. Despite well-known model systems such as upregulation of *Drosophila Cyp6g1*, many putative examples lack functional validation. The potential types of transposon-associated changes that could lead to resistance are reviewed, including changes in up-regulation, message stability, loss of function and alternative splicing. Where potential mechanisms appear absent from the resistance literature examples are drawn from other areas of biology. Finally, ways are suggested in which transgenic expression could be used to validate the biological significance of TE insertion. In the absence of such functional expression studies many examples of the association of TEs and resistance genes therefore remain as correlations.

## 1 Introduction

Transposable elements (TEs) are key drivers of adaptive change and their role in a wide variety of examples of natural selection is well documented. For example, in fish an intronic TE insertion associates with golden coloration in the Midas cichlid (1). In plants, the insertion of two TEs is associated with changes in flower color in commercially grown *Petunias* (2). Within insects themselves the insertion of a TE in the gene *Cortex* (3) is correlated with melanic morphs of the famous Peppered moth, *Biston betularia*, which underlie this classic example of industrial melanism (4). However, despite extensive work on the use of TEs to transform non-model insects (5–8), less functional emphasis has been given to their likely role in adaptation. Moreover, the discovery of potentially TE mediated insect mutants in nature has largely been driven by chance, usually via the directed cloning of genes underlying phenotypes of interest, such as industrial melanism (3). Critically, as many of these examples are from non-model insects, functional proof of the role of TEs in the phenotypes themselves are therefore usually lacking.

Transposable elements can cause a wide variety of genomic changes ranging from simple insertions-deletions to more complex rearrangements such as inversions, duplications or translocations driven by the repetitive sequences they harbor and propagate. Transposons can therefore lead to changes that are in turn reversable such as the *white* eye color mutant of *Drosophila* (9). Similarly, insertion of a *gypsy* element into the *Drosophila yellow* locus causes a reversible color change associated with the *yellow2* or *y2* allele. In this example, homologous recombination between the two long terminal repeats (LTRs) of the *gypsy* element causes reversion to a single LTR that no longer causes a mutant phenotype (10). Transposons themselves seem to represent a sub-population of the genome whose codon usage often differs from that of the host genome (11). However, many of the simple repeats scattered throughout eukaryotic genomes may be the products of the movement of the TEs that inhabit them. In turn these repeats may act to stimulate more complex chromosomal rearrangements such as gene duplication, inversions and translocations. In insects, transposons have been given considerable attention in their potential to genetically transform non-model insects (12, 13) and these classes of common TEs have therefore often been given research priority (14, 15). However different classes of TEs also show dramatically different rates of movement in different strains of animals (16, 17), which may or may not be correlated with the apparent abundance of any given element in any given individual genome. Thus, despite the well characterized P- and M- strains of *Drosophila melanogaster*, which show differing levels of *P*-element activity, inter-strain differences of TE activity amongst other insects remain poorly characterized. This is probably due to the difficulty in maintaining large numbers of strains of non-model insects which directly hinders our ability to characterize rates of TE movement in different strains.

The potential for TEs to specifically cause insecticide resistance was first critically examined by Thomas Wilson (18). Wilson cloned a novel helix-loop-helix transcriptional regulator from *P*-element generated methoprene (a juvenile hormone or ‘JH’ analog) resistant *Drosophila* mutants, termed *Methoprene resistant* or *Met.* These insertions in the *Met* gene generated simple loss of function mutants, which fail to encode a vital component of the subsequently identified multi-component JH receptor. In this study P element mediated mutagenesis was used to identify resistant mutants in order to facilitate cloning of the underlying gene. However, these mutants also highlighted the potential role that TE insertions could play in xenobiotic resistance in general. Despite this prediction, however, 30 years later documented cases of TE mediated insecticide resistance remain rare. Here we critically examine known examples of TEs associated with insecticide resistance. We examine potential changes in transcriptional regulation, message stability, alternative splicing and gene function/loss associated with TE insertions. We also suggest how the biological functions of these insertions can be further validated both in genetic models such as *Drosophila* and in non-model organisms. Finally, we propose some potentially novel mechanisms by which TEs might cause resistance and discuss how likely it is that these would be detected in current studies.

## 2 Up-regulation

### 2.1 Cytochrome P450s as metabolic genes

**Figure 1:**
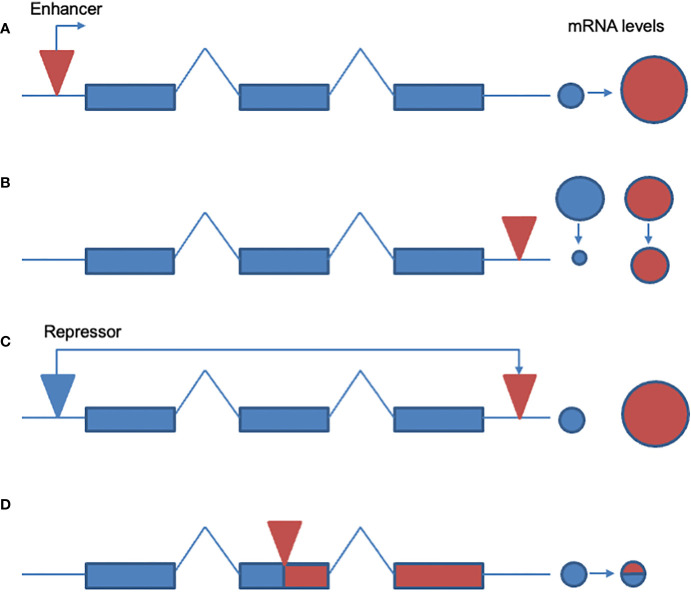
Known and potential mechanisms whereby TEs might cause insecticide resistance. **(A)** Transcriptional up-regulation. A TE insertion in the 5’ end of a potential resistance gene may induce transcriptional upregulation and/or a new pattern of expression of a metabolic gene. An example would be the insertion of the *Accord* LTR into the 5’ end of the *Drosophila Cyp6g1* gene. **(B)** Increased message stability. Insertion of a TE into the 3’ end of the gene increases message stability and leads to the over-expression of a resistance associated gene product. Examples of this type of resistance mechanism have been suggested but not proven (see text for discussion). **(C)** Removal of repressor. TEs might cause the excision and movement of a gene away from a local repressor element therefore leading to upregulation. No documented examples of this potential resistance mechanism exist to date (this panel is therefore not referenced in the text). **(D)** Truncated gene product with novel function. TE insertion disrupts the open reading frame of a gene truncating the associated protein which then adopts a novel function, as speculated for the *CHKov1* gene (see text).

The classic model of transcriptional up-regulation of metabolic activity remains the over-expression of the *Drosophila* cytochrome P450 CYP6G1associated with the insertion of an *Accord* footprint in the 5’ end of the *Cyp6g1* gene. This gene was positionally cloned via classical genetic mapping and with reference to *P* element insertions of known genomic location (19). This rather laborious approach was necessary because the *Resistance to DDT* or *DDT-R* phenotype is dominant and is not uncovered by the classical deficiency mapping widely used in *D. melanogaster* to identify the genes underlying different phenotypes. Preliminary studies suggested that there had been a single insertion and excision of an *Accord* retrotransposon, leaving a long terminal repeat (LTR) 291 bp upstream of the transcription start site (see **Figure 1A** for diagram of this type of mechanism), that was apparently able to increase transcription of the downstream gene *Cyp6g1*.

**Figure 2:**
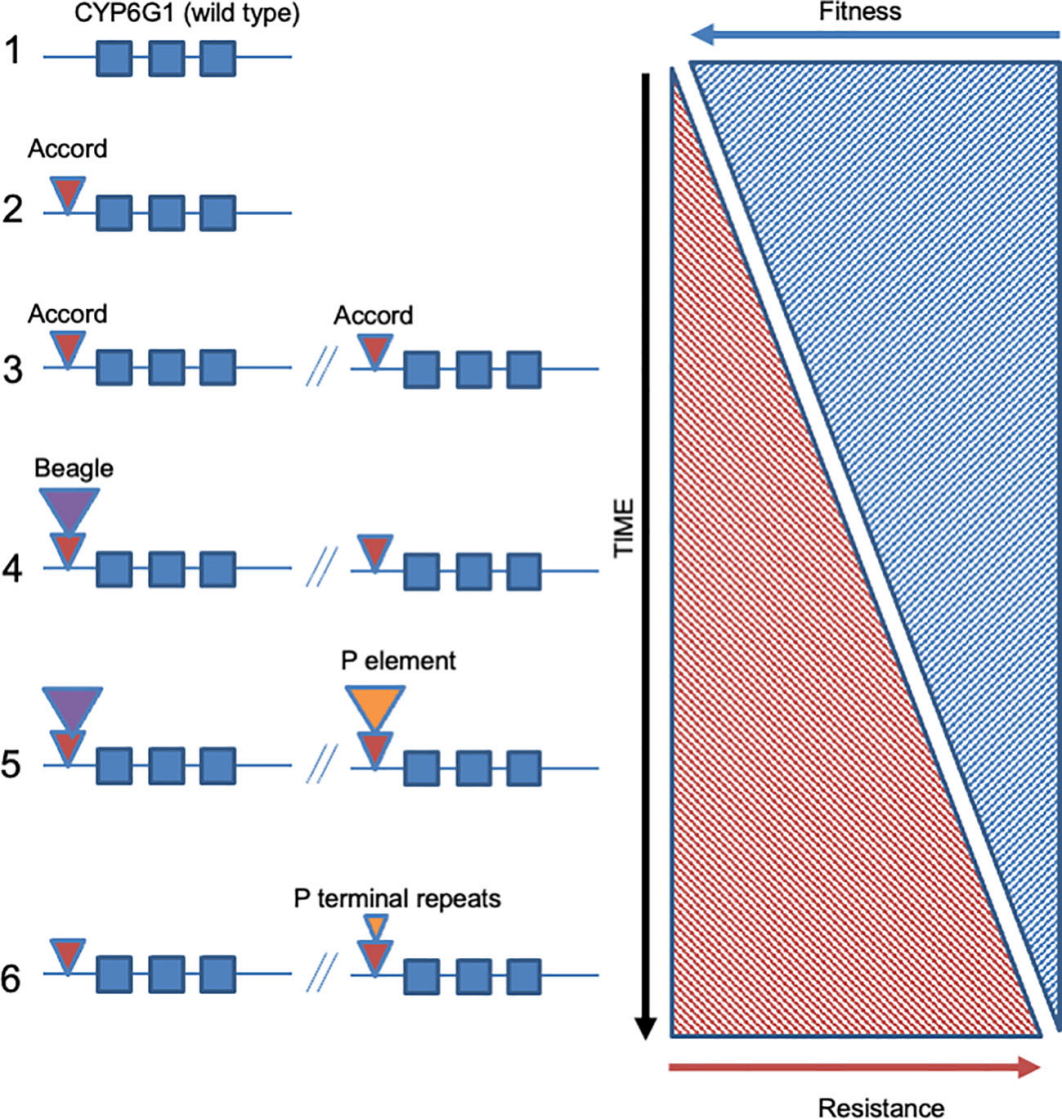
Allelic series shown by TE insertions into the 5’ end of the cytochrome P450 encoding *Drosophila* gene *Cyp6g1*. The six known alleles of *Cyp6g1* are diagramed with the wild type allele at top (panel 1). Allele 2 corresponds to the original insertion of the *Accord* LTR. Allele 3 represents the duplication of the *Cyp6g1* locus. Alleles 4-6 represent further TE insertions into the 5’ ends of both copies of *Cyp6g1*. Note that the TE insertions tend to target the same ‘hot-spot’ and that some are internal to each other (e.g. *Beagle* insertion into the *Accord* LTR in allele 4). The levels of insecticide resistance are thought to increase through the allelic series (from top to bottom) whereas fitness costs associated with each new allele would be expected to decrease. Note that these only represent the extant alleles that have been sequenced and that he presence of further undocumented alleles, new alleles, or now extinct alleles, is likely (see text for discussion).

This insertion upregulates expression of the CYP6G1 enzyme in the midgut, Malpighian tubules and fat body of the fly (20). Genetic transformation of *D. melanogaster* with a copy of *Cyp6g1* under the control of *UAS : GAL4* and a heat shock promoter showed that recombinant over-expression of the wild type copy of this gene was sufficient to confer resistance to DDT. Moreover, transgenic expression of just the *Accord* footprint itself was sufficient to restore the native pattern of expression of the resistance allele in gut and Malpighian tubules of the fly (20). Modelling of the predicted structure of the CYP6G1 enzyme suggests that its unusually shaped active site may allow docking and metabolism of a wide range of insecticides and other xenobiotics (21). In a parallel approach using the *Drosophila* reference panel, *Cyp6g1* was also independently confirmed as a resistance gene by both transcriptomic and genomic associations (22). Parallel studies in the sister species *D. simulans* also showed that *Cyp6g1* was correlated with DDT resistance in this species but in this case the insertion was that of a *Doc* element. Subsequently, the sequencing of more *DDT-R* alleles has in fact showed that this resistance gene has evolved via a classic allelic series, or so called adaptive walk, with numerous different TEs inserting as the same position (23) to give alleles with increasing levels of resistance and presumably decreased fitness costs (**Figure 2**). In this example, the *Cyp6g1* gene is not only duplicated but the *Accord* insertion is joined sequentially via several other TE insertions that appear to further up-regulate P450 expression thereby increasing resistance. These further TEs often insert within the remnants of the previous TE insertion, thereby presumably adding further enhancer sequences, in a manner reminiscent of a Russian doll. The potential mechanisms whereby each allele in this series might decrease fitness costs is however still not clear. Following the discovery that upregulation of *Cyp6g1* can cause insecticide resistance, several studies have examined the potential role of other insect P450 genes as ‘hot-spots’ for TE insertion. For example Chen and co-workers suggested that TE insertions were more frequent in cytochrome P450 genes (24). However, it should be noted that little functional evidence exists for the biological relevance of these potentially P450 oriented insertions and this therefore remains an interesting but unproven correlation (see discussion in conclusions). Finally, it is technically possible that a repressor element for a resistance related gene might be moved (**Figure 1C**) or deleted following TE insertion or deletion. However, this type of mechanism has not been documented to cause insecticide resistance to date.

### 2.2 Target site encoding genes as TE targets

Although it is easier to envisage how TE insertion might up- or down-regulate the transcription of genes encoding enzymes capable of metabolizing xenobiotics, it is technically possible that up- or down-regulation of genes encoding insecticide targets (receptors) might also influence susceptibility. These sort of changes to ion channel gene expression may gain some precedent from studies of genes that interact with *para*, the gene encoding the major subunit of the insect voltage gated ion channel. The *para* gene was originally cloned from a *Drosophila* mutant that showed temperature sensitive paralysis, thus the full gene name *paralytic temperature sensitive* or *para^ts^* (25). Transposable element insertions into the coding sequence of the *para* gene itself are predicted to result in lethality. For example, a P element insertion in the *para^hd5^* mutant allele is lethal in homozygous condition and fly larvae die as early instars (25). However, another temperature sensitive mutant, termed *no action potential temperature sensitive* or *nap^ts^*, identified in a similar screen has been shown to exert changes in *para* gene expression that were proposed to play a role in dosage compensation, as *para* is encoded on the X chromosome and is thus hemizygous in XY male flies. More recent work has shown that *nap^ts^* is an allele of *maleless* or *mle*. The *mle* gene encodes a helicase needed to resolve the complex double stranded RNA (dsRNA) formed during the RNA editing of *para* and encompassing the edited exon and the downstream intron (26). Whilst the toxicological significance of such helicase mutants has not been directly examined, several different point mutations within *Drosophila para* are directly equivalent to both *knockdown resistant*, *kdr*, or *super-kdr* mutants in other insects (27). Up- or down-regulation of *para* itself may, technically, therefore alter the sensitivity of an insect to DDT or pyrethroids by providing either a deficit or excess of target protein in the native voltage gated sodium channel. Similar changes to other voltage- or ligand-gated ion channel targets may also affect their insecticide sensitivity in currently undocumented ways. Finally, it should be noted that not all TE insertions into the coding sequence of insecticide receptors are in fact lethal. For example, several mutations in subunit 6 of the insect nicotinic acetylcholine receptor have been reported that lead to non-functional or truncated proteins associated with spinosyn resistance (28–30). So, in conclusion, if the insecticide binds to a receptor subunit that is somehow dispensable (not associated with lethality) then further direct TE insertions into the open reading frames of resistance genes remain a possibility.

## 3 Message stability disrupted by TEs

As well as causing up- or down-regulation of enzymes capable of metabolizing or sequestering insecticides, TE insertions have also been correlated with apparent changes in message stability in insects (see **Figure 1B** for diagram of this potential type of mechanism). Where these messages encode enzymes such as cytochrome P450s that are capable of metabolizing xenobiotics such changes may lead to the corresponding over-expression of the gene product and subsequent resistance. A potentially interesting example of this, beyond *Cyp6g1*, is the presence of a *Bari1* insertion in the 3’ end of the *Cyp12a4* cytochrome P450 gene. Although the transcript of the truncated gene only contains 18 base pairs of TE sequence, it is also ten-fold overrepresented in flies carrying the insertion. This TE insertion was identified because it is fixed in a wide variety of field and laboratory *Drosophila* strains (31). However, the biological relevance of this insertion and indeed the likely substrates of the P450 CYP12A4, native or xenobiotic, remain unknown. Similarly, potential changes in the length and stability of the *Cyp6a2* mRNA were originally proposed to explain *DDT-R* in *91-C* (susceptible) and *91-R* (resistant) strains of *D. melanogaster* (32). In this example, the susceptible *Cyp6a2* allele carried a single LTR of a TE in its 3’ end which was suggested, but not proven, to change *Cyp6a2* message stability. However, given that these changes are in the susceptible, and not resistant, allele of this gene it remains unclear what its potential role in resistance might be. Further, a subsequent study found no correlation between the presence of this LTR and resistance (33), suggesting that it was indeed only a coincidence based on a limited sample size.

We note that such potential changes in message stability are particularly hard to prove and may indeed still be correlated with undescribed changes to the 5’ end of the associated genes and in fact could therefore still be further examples of transcriptional upregulation. Proving the potential of these insertions to increase or decrease message stability would require detailed studies of the half-lives of the different transcripts *in vivo*, studies which to date are generally lacking. Despite the lack of functional evidence for changes in message stability in insects, important examples of this process in other animals can be readily found. For example, insertion of a B2 short-interspersed repeat (termed B2 SINE) into the 3’ end of the mouse catechol-O-methyltransferase (*COMT*) gene introduces a premature polyadenylation signal, creating a short untranslated region (UTR) 3’ isoform (34). As COMT is a key enzyme responsible for degradation of dopamine and norepinephrine, changes in mRNA and protein abundance lead to key changes in synaptic function and associated behaviors (34). Changes in message stability associated with TEs therefore remain an interesting but largely unproven potential mechanism of insecticide resistance

## 4 Alternative splicing and TEs

Theory dictates that ectopic recombination between different TE insertions can scramble chromosomes and therefore that under most circumstances numerous insertions are harmful (35, 36). Given this simple expectation, insertions such as those in the 5’ end of *Cyp6g1*, which are unusually frequent in *D. melanogaster* populations, support the idea that the gene is under strong selection via the unexpectedly wide cross-resistance associated with the *DDT-R* phenotype. Similarly, it is possible to survey TE insertions in a range of insect strains and then to try and establish what selective force any abundant insertions might be under. This approach was used by Aminetzach and co-workers (37) to examine 16 identified insertions of *Doc* transposons in 100 different *D. melanogaster* strains. One element, *Doc1420*, was found at unexpectedly high frequency, except in a putatively ancestral populations of flies from central Africa, and seems to be associated with, or closely linked to, a recent selective sweep. The insertion, into the second exon of a gene they termed *CHKov1*, causes complex changes in alternative splicing of the mutated transcripts (see **Figure 1D** for diagram illustrating this type of potential mechanism), none of which contain all four exons of the wild-type gene. Further analysis of this fascinating example is complicated by the unknown function of the *CHKov1* gene product. However, based on limited predicted amino acid homology to a putative choline kinase, the authors suggest that CHKov1 is involved in choline metabolism. Further, they speculate that changes in expression of this gene may therefore affect the insecticide target-site acetylcholinesterase, the enzyme that degrades the neurotransmitter acetylcholine within the synaptic cleft. Finally, they showed that four mutant fly lines showed resistance to the insecticide azinphos-methyl-phosphate which is an acetylcholinesterase inhibitor. Whilst this study clearly needs to be validated by transformation of the truncated *CHKov1* gene into susceptible flies to demonstrate that it can indeed confer resistance, this study raises the formal possibility that truncated gene products resulting from TE associated changes in alternative splicing can cause resistance. This type of mechanism, whereby an insertion of a TE causes a novel function for the targeted gene product is well known from other examples of insect adaptation. For example, recent surveys of resistance to *Drosophila* virus A in *Drosophila melanogaster* have found that viral resistance is associated with the insertion of a *Doc* element into the open reading frame of the gene *Veneno* (38). In this context it is important to note that the original gene product has no known role in virus resistance and therefore the inference is that the TE truncated *Veneno* gene product gains a new function in protecting against infection. This gain of function following TE insertion seems rare in insecticide resistance, but this is clearly a mechanism to watch for in the future.

Although perhaps much harder to imagine, changes in alternative splicing might also render ion channel insecticide targets resistant to insecticide binding. Despite the recent growth in insecticidal protein toxins, ion channels remain as the most important target sites for the major classes of current insecticides (39) and many of the genes encoding these different channel subunits show alternative splicing (40). Where such alternative splicing is extensive it is not hard to imagine how changes in the representation or length of different isoforms might alter the insecticide sensitivity of the resulting ion channel. However, this situation is substantially complicated by our striking lack of understanding of the exact native subunit composition of any insect ion channel. For example, the target site of DDT and pyrethroid insecticides is the voltage gated sodium channel of which the major subunit is encoded by the *temperature sensitive paralytic* or *para^ts^* gene, discussed above in the context of changes in channel density. The *para* gene shows extremely complicated alternative splicing, involving the potential use of alternative exons leading to over 48 splice forms (41) which appear to be conserved in other species (42). Whilst the relative affinity of these different splice forms for pyrethroids has not been directly tested via functional expression, disruption of these alternative exons and/or their acceptor or donor sites might result in isoforms with altered insecticide sensitivity. Similarly, the chloride ion channel that is the target of cyclodiene and fipronil insecticides contains subunits encoded by the gene *Resistance to dieldrin* or *Rdl*. The *Rdl* gene also displays alternative splicing (40) and isoforms differ in their sensitivity to the agonist, gamma-aminobutyric acid or GABA (43). Whilst cyclodiene-like insecticides interact with residues in the second membrane spanning region (M2) of the encoded RDL subunit, a region thought to line the integral chloride ion channel, these insecticides bind preferentially to the desensitized state of the receptor via allosteric effects (44). It is therefore not inconceivable that changes in GABA sensitivity, associated with TE mediated isoform disruption, could in turn alter the amount of time the receptor spends in this drug preferred desensitized state and thus alter drug sensitivity itself.

In conclusion, TE driven changes in alternative splicing remain an attractive, but poorly documented, method of likely insecticide resistance. This situation is further complicated by our central lack of understanding of the true subunit composition of the native receptors that contain *para* or *Rdl* encoded subunits, rendering accurate predictions difficult. For example, despite the cloning of the auxiliary voltage gated sodium channel subunit TipE in 1995 (45), and the high levels of conservation of a group of TipE-like subunit encoding genes in the *Drosophila* genome (46), the toxicological relevance of this accessory subunit still remains obscure (47). Similarly, despite the fact that much of the pharmacology of native insect GABA receptors can be reconstituted by the functional expression of RDL homo-multimers and despite the widespread expression of RDL in the insect nervous system (48) the toxicological role of other GABA receptor-like subunits (GABA/glycine like receptor of *Drosophila* or GRD and Ligand-gated chloride ion channel homologue 3 or LCCH3) encoded in insect genomes (49) and their ability to co-assemble with RDL *in vitro* remain poorly characterized (50). Finally, whilst the focus of this review is largely on examples drawn from resistance to small molecule pesticides, it is worth noting that mis-splicing of a gene encoding the cadherin associated with resistance to the *Bacillus thuringiensis* (Bt) toxin Cry1Ac has been shown in the pink bollworm. In this example, a miniature inverted repeat transposable element (MITE), also carrying two additional TEs, produces two mis-splice transcript variants of the cadherin receptor (51). Strains homozygous for this insertion not only confer 290-fold resistance to Cry1Ac but can complete their full lifecycle on transgenic cotton expressing the Cry1Ac toxin (51). In this case, therefore, the homozygous TE insertion does not appear lethal (see below for further discussion of lethality).

## 5 Loss or gain of susceptible receptor subunits

**Figure 3:**
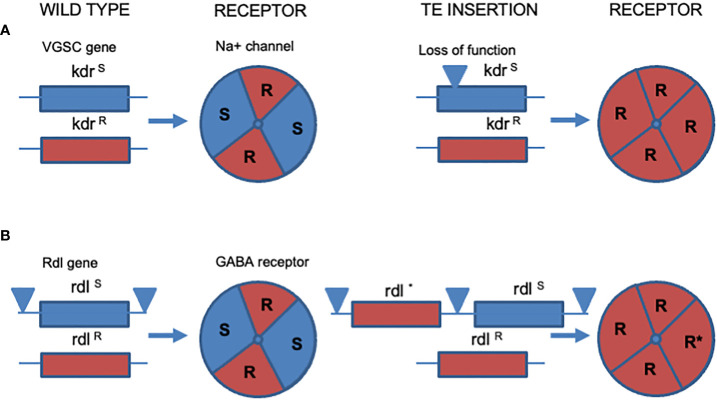
Loss or maintenance of receptor subunits associated with resistance. **(A)** The insect Voltage Gated Sodium Channel (VGSC) encoded by the *knockdown resistance* (*kdr*) gene. An insect heterozygous for *kdr* resistance, *kdrR/kdrS* (left hand panel) suffers a TE insertion into the susceptible allele which stops expression of the corresponding susceptible receptor VGSC subunit (right hand panel). The corresponding receptor is therefore changed from one carrying both R and S subunits (left) to one containing only resistant subunits. In this manner an allele that is recessive (kdrR) is uncovered and becomes fully dominant in its newfound hemizygous condition (*kdrR/-*). **(B)** Duplication and maintenance of a susceptible insect gamma-amino butyric acid receptor (GABA-R) encoded by the *Resistance to dieldrin* (*Rdl*) gene. In an insect heterozygous for resistance (*RdlR/RdlS*) transposons flank the susceptible copy of the *Rdl* gene (left panel). The flanking transposons cause duplication of the *RdlS* gene and subsequent mutation of one copy to *RdlR* (right panel). The resulting compound genotype (*RdlRS/RdlR*) therefore always encodes susceptible copies of the RDL GABA receptor, potentially offsetting any biophysical deficits associated with native receptors carrying only drug insensitive subunits (see text for discussion).

Loss of function mutants, where TE insertion disrupts a functional open reading frame, can unexpectedly cause resistance if the remaining receptor/ion channel encoding allele is resistant. In a striking example of this, knockout of a susceptible copy of *kdr* (see TE insertion diagrammed in **Figure 3A** for example) in a heterozygous but otherwise recessive resistant aphid clone rendered the clone fully resistant by placing the recessive resistant copy of the *para* gene into a hemizygous state (52). If such an aphid clone does not go through sexual reproduction it may persist in this hemizygous condition where all the voltage gated sodium channels encoded in its nervous system are therefore resistant to the effects of pyrethroid insecticides (**Figure 3A**), despite normally being recessive in the presence of their susceptible receptor subunit counterparts. Such ‘orphan’ (hemizygous) resistance alleles might be expected to carry a fitness cost as the wild type functions carried by the susceptible receptor subunits are also lost. Recent studies have however highlighted that these effects can also be offset by within allele duplications that maintain both susceptible and resistant copies on the same chromatid. For example, the *Rdl* gene can be found as duplicated alleles where two copies of the receptor encoding gene are found side-by-side (as diagrammed in **Figure 3B**), one resistant and one susceptible (53). This ‘enforced’ (compound) heterozygosity allows for the persistence of one wildtype (susceptible) copy of the *Rdl* subunit encoding gene and therefore facilitate the persistence of susceptible RDL subunits in the associated native receptor (**Figure 3B**). Thus, maintenance of wild-type receptor subunits will therefore potentially offset any fitness costs associated with receptors composed only of resistant subunits (39, 54). These findings are not restricted to resistance to small molecule insecticides but also parallel TE insertions conferring resistance to *Bacillus thuringiensis* or Bt toxins, where resistant individuals can carry alleles with the same or different mutations, as discussed by Panini and others (52).

## 6 Genomic rearrangements and TEs

Although any role of TEs in the duplication of such target site encoding genes has not been shown, it is highly likely that such duplications are facilitated by duplicated sequences found either side of the parent gene. Given the likely age of such duplications it is not surprising that the mode of their duplication is not immediately obvious but repeated sequences associated with TEs are likely candidates both for their origins and indeed their potential subsequent loss. Examples of gene duplication leading to the over-expression of metabolic genes are widespread. But again, the age of these duplication events tends to obscure the mechanisms of their origin. Moreover, current sequencing technologies tend to be very good at resolving the structural genes involved in such duplications but less good at resolving the repeated sequences that may flank these blocks of duplications. Ironically, we are therefore left unable to see the likely culprits responsible for the compilation of the flanking repeats themselves but TEs remain as prime candidates.

## 7 A likely role for epigenetics

Recent evidence suggests that epigenetics also plays a key role in the potential evolution of insecticide resistance. The most obvious mechanism by which this could occur is via changes in DNA methylation in subsequent generations following insecticide exposure (55). Critically, however, DNA methylation is often detected by genome wide bisulfide sequencing and little attention is paid to the exact site of differential methylation. Preferential methylation of TEs or their associated sequences is therefore an interesting possibility for the evolution of resistance, particularly given their propensity to jump into the 5’ end of resistance genes whose upregulation via differential methylation then becomes likely. In this light, examples of insecticide reversion and re-selection in the Peach Potato aphid *Myzus persicae*, shown to involve changes in DNA methylation (56), would be worth re-investigating to see if differential methylation is TE associated or not. Similarly, there has been considerable speculation as to whether an insect’s diet can pre-adapt it to become resistant to a xenobiotic (57). Whilst this remains a controversial topic, likely mechanisms of detoxifying enzyme up-regulation have not been investigated and again different methylation of TE associated sequences remains a good candidate. Finally, changes in methylation might also be involved in the changes in metabolic activity observed in insect gut bacteria under insecticide selection (58). This reminds us that we need to look at changes in the holo-genome of insects where the host genome and those of its microbial symbionts are looked at together.

## 8 The causal nature of TE insertions

As is clear from the above discussion, many potential examples of TEs and resistance are simple observations of the presence of a TE related sequence in or near a gene of interest. Therefore, functional validation of the role of the TE related sequence often remains to be performed. Although supporting evidence for the functional role of TE insertion can come from population genetic analyses showing peaks of selection around the insertion, proving that a given sequence leads to the expected change in a non-model organism is far from simple. In the unusual case of *Cyp6g1* it was possible to splice the *Accord* footprint onto a reporter gene and prove that this sequence alone was necessary and sufficient to reconstitute the native pattern of transcription of the mutant gene in the *D. melanogaster* gut. The challenge of proving that other TEs or their associated sequences drive similar changes in expression patterns is complicated by the fact that TEs themselves have their own patterns of expressions within the insects within which they are found. This raises an interesting conundrum as it suggests not only that some genes may act as ‘hot-spots’ for TE insertion but also that the elements themselves may be further selected for those causing suitable patterns of expression. In the case of *Accord* and *Cyp6g1* this would require not only that *Cyp6g1* is a ‘hot-spot’ for insertion but that the recruited elements also drive expression of the mutant transcript in the gut and Malpighian tubules where most insecticide metabolism is enhanced.

## 9 Conclusions and the future

This review has highlighted known mechanisms whereby TE insertion can cause insecticide resistance both to small molecule insecticides and to insecticidal toxins. It has also highlighted potential mechanisms found in other cases of TE mediated adaptation that have not yet been found to be associated with insecticide resistance. As the number of sequenced insect genomes continue to grow, we can also begin to survey TEs across multiple genomes and to ask if specific subsets of genes are targeted by TE insertion. To this end a recent survey of 21 *Myzus persicae* genomes has shown certain classes of metabolic enzymes, such as the cytochrome P450 encoding genes, to be enriched for TE insertions (59). More comparative genomic analyses should therefore provide us with multiple potential TE insertions which can then be in turn be tested for their functional validity. Genomics therefore promises to vastly increase the number of potential insecticide resistance associated TE insertions but the difficulty of functionally testing each insertion in non-model insects remains.

## Author contributions

The author confirms being the sole contributor of this work and has approved it for publication.
